# Bible Confirmations

**Published:** 1879-03

**Authors:** 


### BIBLE CONFIRMATIONS.

FOUR thousand years ago was built the Temple of Karnak, covered inside and out with hieroglyphics detailing the histories of the passing ages, making the official record of successive dynasties, their victories and their defeats, their conquests and their crosses. But for thousands of years this Temple was in ruins, its walls were fallen in; its capitals, its cornices, and its columns, all crumbled, broken and defaced; and for these same thousands of years, these records were a closed volume; no man was found to "read the writing" up to the beginning of the present century. Just previous to that time, the expedition to Egypt under Napoleon the First, gathered together a vast number of valuable antiquities, among which was a broken black stone, some three feet by two, basaltic; and on this, the famous Rosetta stone, were three lines, one was in hieroglyphics, one in the hand-writing, as it were, of the common people, and one in Greek. The great Champolion conjectured that they read the same thing, as did the famed words in Greek and Hebrew and Latin, at the Crucifixion; by this key, the immortal Frenchman read the hieroglyphics of Karnak. But of all the lines on Karnak's walls, one spot alone remained entire, giving the record of a Bible event. The moment his eye fell upon it (singled out, as it were, the only whole among a million of others in ruins), he was filled with amazement, as he mechanically exclaimed.

*Melek Aiuda*, or King of Judah; thus offering a magnificent tribute to the truthfulness of God's Revelation, in that, at a point in the long ages of the past, there was a country, that it was under a form of kingly government, and that it had a name, "The King of Jews," thus linking together Jesus and the Cross, the Jews and Judah and Shishak, the very name of the Kingdom of Solomon, as one connected history. A few years later, and those lines on Karnak's walls began to fail and fall, and are now no longer legible, except in part. But while they were legible, fac-simile copies were taken and scattered among the libraries of the world, to stand for all time to come as witnesses of God's truth, as if they were preserved just long enough for the world to read them, and then be blotted out forever! Thus it is that the Almighty takes care of the Bible and its truths.

